# Metformin suppresses pediatric acute myeloid leukemia cell viability and clonogenicity

**DOI:** 10.1186/2049-3002-2-S1-P23

**Published:** 2014-05-28

**Authors:** Anilkumar Gopalakrishnapillai, E Anders Kolb, Sonali Barwe

**Affiliations:** 1 Alfred I duPont Hospital for Children, Wilmington, DE, USA

## Background

Acute myeloid leukemia (AML) accounts for 15-20% of childhood leukemias. Although remission is achieved following treatment with front-line chemotherapy, nearly half of the patients are faced with disease relapse associated with chemoresistance. Therefore, therapies that could sustain the remission phase in pediatric AML are urgently needed. Metformin is a widely used anti-diabetic drug known to exhibit anti-cancer effect in several malignancies. Metformin is well-tolerated at very high doses, and its safety has also been demonstrated in clinical trials in diabetic children.

## Materials and methods

Cell viability was estimated using the Cell Titer Blue assay. Cells were stained with Annexin V-FITC and apoptotic cells were detected by flow cytometry. The activation status of AMP-activated protein kinase (AMPK) was detected by immunoblotting for phosphorylated (T172) AMPKα subunit. Metformin-treated cells were plated in methylcellulose complete medium containing metformin for clonogenicity assay. The number of colonies were counted after 15 days. The AML xenograft model was generated by inoculating metformin-treated cells into NSG-B2m mice via the tail vein. The disease progression was monitored by flow cytometry.

## Results

Treatment with metformin decreased the viability of several AML cell lines in a dose-dependent manner with IC50 ranging from 1.57 to 12.65 mM. IC50s were reduced when metformin treatment was performed in low glucose medium or in the presence of a glucose uptake inhibitor fasentin. Metformin induced G1 cell cycle arrest and apoptosis in these cells. Treatment with low doses of metformin (0.5 mM) resulted in suppression of colony formation in a clonogenicity assay. In correlation with this data, we observed that low dose metformin treated cells exhibited delayed engraftment in an AML xenograft mouse model with 50% reduction in the percentage of AML cells in mouse blood. Metformin is known to induce AMPK activation in a variety of cancer cells. This AMPK activation has also been shown to be essential for metformin-induced cell death in certain cell types. Metformin caused AMPK activation in MV4;11 and NB4 cells, but failed to induce AMPK phosphorylation in THP-1 cells, indicating that AMPK activation by metformin is cell-type specific.

## Conclusions

Metformin induced G1 cell cycle arrest and apoptosis in AML cells. At low doses, metformin suppressed clonal growth and delayed engraftment in the AML xenograft mouse model. Differential activation of AMPK by metformin in different cell lines suggests a possible AMPK-dependent or independent mechanism of metformin-induced cell death.

